# (*E*)-5-[1-Hy­droxy-3-(3,4,5-tri­meth­oxy­phen­yl)allyl­idene]-1,3-di­methyl­pyrimidine-2,4,6-trione: crystal structure and Hirshfeld surface analysis

**DOI:** 10.1107/S2056989017010374

**Published:** 2017-07-18

**Authors:** Mónica Soto-Monsalve, Elkin L. Romero, Fabio Zuluaga, Manuel N. Chaur, Richard F. D’Vries

**Affiliations:** a Instituto de Química de São Carlos, Universidade de São Paulo 13566-590, São Carlos, Brazil; b Departamento de Química, Universidad del Valle, AA 25360, Cali, Colombia; c Universidad Santiago de Cali, Calle 5 # 62-00, Cali, Colombia

**Keywords:** crystal structure, Hirshfeld surface, supra­molecular structure, allyl­idene

## Abstract

In the title compound, C_18_H_20_N_2_O_7_, the dihedral angle between the aromatic rings is 7.28 (7)° and the almost planar conformation of the mol­ecule is supported by an intra­molecular O—H⋯O hydrogen bond, which closes an *S*(6) ring. In the crystal, weak C—H⋯O hydrogen bonds and aromatic π–π stacking link the mol­ecules into a three-dimensional network. A Hirshfeld surface analysis showed that the major contribution to the inter­molecular inter­actions are van der Waals inter­actions (H⋯H contacts), accounting for 48.4% of the surface.

## Abstract 2

In the title compound, C—H⋯O hydrogen bonds and aromatic π–π stacking combine to generate a three-dimensional network. A Hirshfeld surface analysis is presented.

## Chemical context   

Bartituric acid derivatives are of inter­est due to their potential biological applications (Bojarski *et al.*, 1985 ▸; Patrick, 2009 ▸). These compounds have materials science appplications due to the properties generated by π-conjugation, such as push–pull chromophores (Klikar *et al.*, 2013 ▸; Seifert *et al.*, 2012 ▸). The chemical structures of these derivatives show five potential metal-binding sites, which makes them versatile ligands for the construction of coordination and supra­molecular compounds (Mahmudov *et al.*, 2014 ▸), also important in organic synthesis, where they are largely used as substrates for Morita–Baylis–Hilmann and Diels–Alder reactions (Goswami & Das, 2009 ▸). Herein we report the crystal structure and Hisrshfeld surface analysis of (*E)*-5-[1-hy­droxy-3-(3,4,5-tri­meth­oxy­phen­yl)allyl­idene]-1,3-di­methyl­pyrimidine-2,4,6-trione (I), which presents potential applications in the study of the photophysical properties of different isomers for the development of supra­molecular structures.
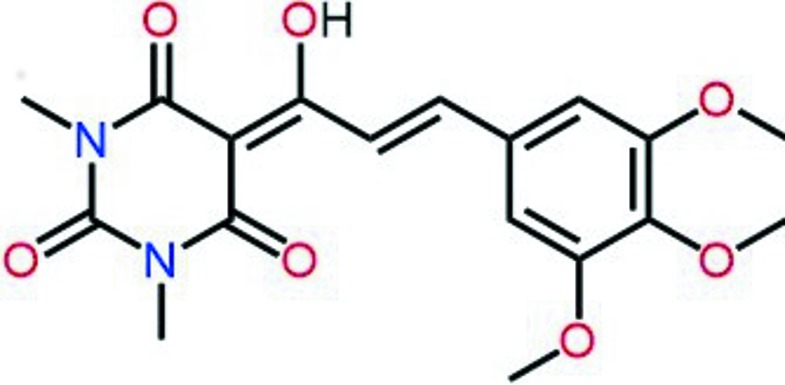


## Structural commentary   

**Figure 1:**
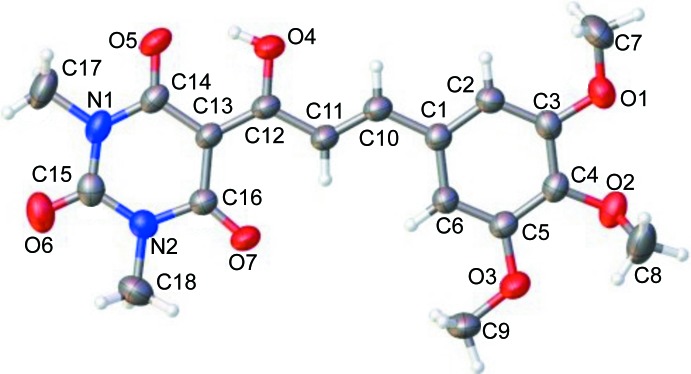
The mol­ecular structure of (I), showing 50% probability displacement ellipsoids.

The structure of (I), which crystallizes in the triclinic space group *P* 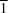, presents conjugation over the C1—C10—C11—C12—C13 bonds, leading to a an almost planar conformation (Fig. 1 ▸); the C10—C11—C12—C13 and C1—C10—C11—C12 torsion angles are −176.76 (1) and −179.27 (1)°, respectively. The dihedral angle between the aromatic rings is 7.28 (7)°. The C atoms of the *meta*-meth­oxy groups lie close to the plane of their attached ring [deviations for atoms C7 and C9 of 0.289 (2) and 0.131 (2)Å, respectively], whereas the *para*-meth­oxy C atom deviates significantly, by 0.959 (2) Å, which is reflected in the C3—C4—O2—C8 torsion angle of 106.41 (19)°. An intra­molecular O—H⋯O hydrogen bond (Table 1 ▸) closes an *S*(6) ring and a C—H⋯O inter­action is also observed. A *Mogul* geometry check found that all the bond lengths and angles are within typical ranges (Bruno *et al.*, 2004 ▸).

## Supra­molecular features   

**Figure 2:**
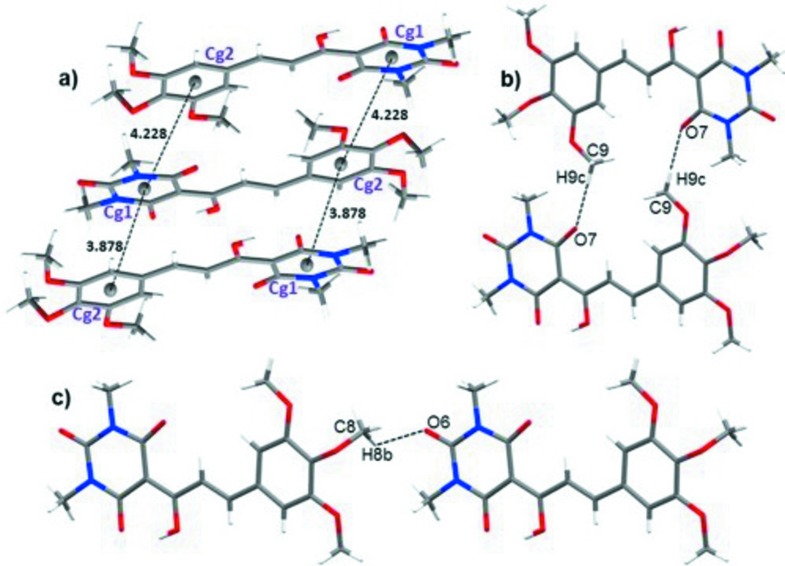
Details of the inter­molecular inter­actions in the crystal of (I), showing (*a*) π–π stacking between rings 1 (N1/N2/C13–C16) and 2 (C1–C6) along the [010] direction, (*b*) an inversion dimer formed by the C9—H9*C*⋯O7^ii^ hydrogen bond and (*c*) by the C8—H8*B*⋯O6^i^ hydrogen bond. [Symmetry codes: (i) *x*, *y*, 1 + *z*; (ii) −1 − *x*, 1 − *y*, −*z*.]

**Figure 3:**
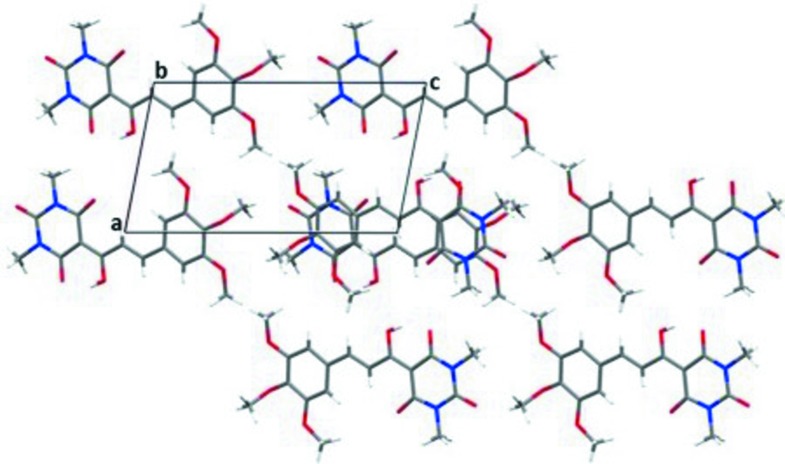
Crystal packing representation of (I), viewed along the [010] direction.

The packing of the title compound features inversion dimers linked by pairs of C9—H9*C*⋯O7^ii^ hydrogen bonds [C⋯O = 3.3694 (19) Å], which generate 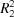
(24) loops. The dimers are linked along the [001] direction by the C8—H8*B*⋯O6^i^ hydrogen bond [C⋯O = 3.341 (3) Å]. In addition, weak and very weak π–π inter­actions (which alternate with respect to the [010] direction) between benzene and pyrimidine rings [centroid–centroid separations = 3.8779 (1) and 4.2283 (9) Å, respectively] occur (Fig. 2 ▸). Together, these inter­molecular inter­actions lead to a three-dimensional network (Fig. 3 ▸).

## Hirshfeld surfaces analysis   

**Figure 4:**
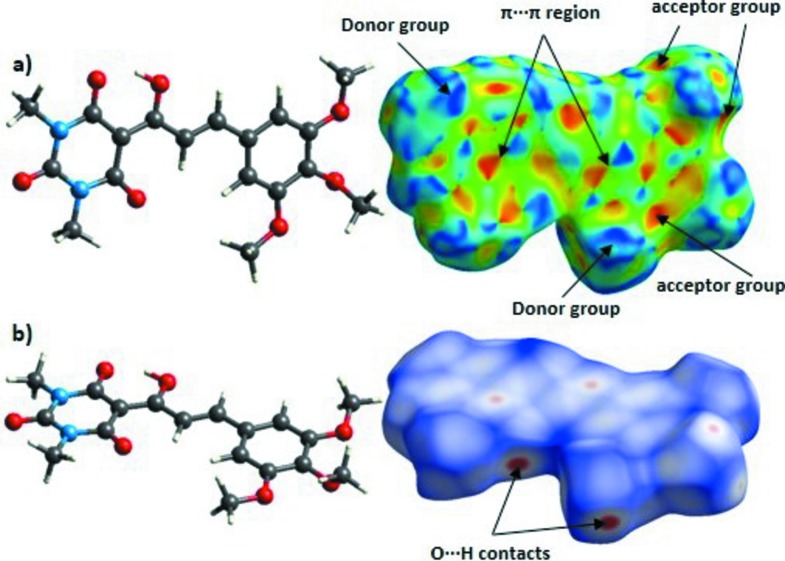
Hirshfeld surface of the title compound as (*a*) shape index and (*b*) *d*_norm_.

The Hirshfeld surface analysis shows the potential inter­molecular contacts. Convex blue regions represent hydrogen-donor groups and concave red regions represent hydrogen-acceptor groups (Hirshfeld, 1977 ▸; McKinnon *et al.*, 2004 ▸). In this case, the main donor groups are the methyl groups and the acceptor groups are the O atoms. The region of π–π inter­actions, observed as red and blue triangles over the aromatic rings, is also clear (Fig. 4 ▸). This surface confirms the importance of the inter­actions described previously.

**Figure 5:**
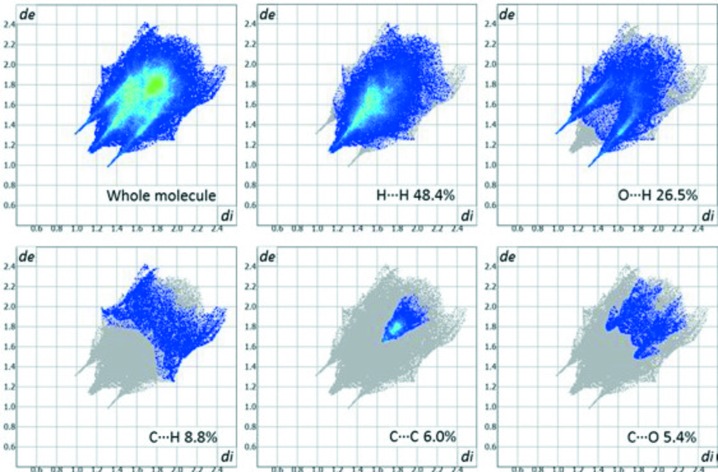
Bidimensional fingerprint plots for the whole mol­ecule and H⋯H, O⋯H, C⋯H, C⋯C and C⋯O close contacts.

The two-dimensional fingerprint plot qu­anti­fies the contribution of each kind of inter­action to the surface formation (McKinnon *et al.*, 2007 ▸). For the title compound (Fig. 5 ▸), the major contribution is due to H⋯H corresponding to van der Waals inter­actions with 48.4% of the surface, followed by the O⋯H inter­action, which contributes 26.5% (this contribution is observed as two sharp peaks in the plot); this behaviour is usual for strong hydrogen bonds (Spackman & McKinnon, 2002 ▸). Finally, π–π inter­actions represented by C⋯C inter­actions contribute 6.0% to the Hirshfeld surface.

## Database survey   

A general search in the Cambridge Structural Database (Groom *et al.*, 2016 ▸) for barbituric acid derivatives yielded 718 hits. Limiting the search for a barbituric acid substituted at position C5 with a phenyl­propyl group yielded 14 hits; two of these results present double-bond conjugation, namely 1,3-dibutyl-5-{3-[4-(di­methyl­amino)­phen­yl]prop-2-en-1-yl­idene}pyrimidine-2,4,6(1*H*,3*H*,5*H*)-trione (Klikar *et al.*, 2013 ▸) and 5-{3-[4-(di­methyl­amino)­phen­yl]prop-2-en-1-yl­idene}pyrimidine-2,4,6(1*H*,3*H*,5*H*)-trione (Seifert *et al.*, 2012 ▸).

## Synthesis and crystallization   

The title compound was prepared according to the literature procedure of Gorovoy *et al.* (2014 ▸). A mixture of 3,4,5-tri­meth­oxy­benzaldehyde and 5-acetyl-1,3-di­methyl­barbituric acid was melted at 453 K and 2–3 drops of piperidine were added under constant stirring. After 5 min, the mixture solidified, providing a yellow powder, which was allowed to cool to room temperature. The solid residue was boiled in ethanol (20 ml) for a few minutes and the precipitate was filtered off by vacuum suction. The filtrate was left at room temperature, yielding yellow needles of the title compound after three weeks.

## Refinement   

Crystal data, data collection and structure refinement details are summarized in Table 2 ▸. H-atom positions were calculated geometrically and refined using the riding model, with O—H = 0.82 Å, methyl C—H = 0.96 Å and aromatic C—H = 0.93 Å.

## Supplementary Material

Crystal structure: contains datablock(s) I. DOI: 10.1107/S2056989017010374/hb7681sup1.cif

Structure factors: contains datablock(s) I. DOI: 10.1107/S2056989017010374/hb7681Isup2.hkl

CCDC reference: 1562022

Additional supporting information:  crystallographic information; 3D view; checkCIF report

## Figures and Tables

**Table 1:** Hydrogen-bond geometry (Å, °)

*D*—H⋯*A*	*D*—H	H⋯*A*	*D*⋯*A*	*D*—H⋯*A*
O4—H4⋯O5	0.82	1.74	2.4841 (15)	150
C11—H11⋯O7	0.93	2.16	2.8044 (18)	125
C8—H8*B*⋯O6^i^	0.96	2.60	3.341 (3)	135
C9—H9*C*⋯O7^ii^	0.96	2.42	3.3694 (19)	170

Symmetry codes: (i) 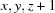; (ii) 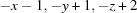.

**Table 2:** Experimental details

Crystal data	
Chemical formula	C_18_H_20_N_2_O_7_
*M*_r_	376.36
Crystal system, space group	Triclinic, *P* 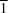
Temperature (K)	296
*a*, *b*, *c* (Å)	7.9989 (3), 8.0659 (3), 14.6533 (5)
α, β, γ (°)	104.520 (1), 98.422 (1), 98.909 (1)
*V* (Å^3^)	887.04 (6)
*Z*	2
Radiation type	Mo *K*α
μ (mm^−1^)	0.11
Crystal size (mm)	1.07 × 0.33 × 0.28

Data collection	
Diffractometer	Bruker APEXII CCD
Absorption correction	Multi-scan (*SADABS*; Bruker, 2015 ▸)
*T*_min_, *T*_max_	0.714, 0.745
No. of measured, independent and observed [*I* > 2σ(*I*)] reflections	26167, 3626, 3176
*R*_int_	0.021
(sin θ/λ)_max_ (Å^−1^)	0.627

Refinement	
*R*[*F* ^2^ > 2σ(*F* ^2^)], *wR*(*F* ^2^), *S*	0.043, 0.130, 1.04
No. of reflections	3626
No. of parameters	250
H-atom treatment	H-atom parameters constrained
Δρ_max_, Δρ_min_ (e Å^−3^)	0.26, −0.22

Computer programs: *APEX2* and *SAINT* (Bruker, 2012 ▸), *SHELXS2013* (Sheldrick, 2008 ▸), *SHELXL2014* (Sheldrick, 2015 ▸), *ORTEP-3 for Windows* (Farrugia, 2012 ▸), *Mercury* (Macrae *et al.*, 2008 ▸), *CrystalExplorer* (McKinnon *et al.*, 2004 ▸), *WinGX* (Farrugia, 2012 ▸) and *OLEX2* (Dolomanov *et al.*, 2009 ▸).

## supplementary crystallographic information

### Crystal data

**Table:**

C_18_H_20_N_2_O_7_	*Z* = 2
*M**_r_* = 376.36	*F*(000) = 396
Triclinic, *P*1	*D*_x_ = 1.409 Mg m^−^^3^
*a* = 7.9989 (3) Å	Mo *K*α radiation, λ = 0.71073 Å
*b* = 8.0659 (3) Å	Cell parameters from 9895 reflections
*c* = 14.6533 (5) Å	θ = 2.6–26.4°
α = 104.520 (1)°	µ = 0.11 mm^−^^1^
β = 98.422 (1)°	*T* = 296 K
γ = 98.909 (1)°	Needle, light yellow
*V* = 887.04 (6) Å^3^	1.07 × 0.33 × 0.28 mm

### Data collection

**Table:**

Bruker APEXII CCD diffractometer	3626 independent reflections
Radiation source: microfocus sealed X-ray tube	3176 reflections with *I* > 2σ(*I*)
Detector resolution: 7.9 pixels mm^-1^	*R*_int_ = 0.021
φ and ω scans	θ_max_ = 26.5°, θ_min_ = 1.5°
Absorption correction: multi-scan (SADABS; Bruker, 2015)	*h* = −10→9
*T*_min_ = 0.714, *T*_max_ = 0.745	*k* = −10→10
26167 measured reflections	*l* = −18→18

### Refinement

**Table:**

Refinement on *F*^2^	Primary atom site location: structure-invariant direct methods
Least-squares matrix: full	Hydrogen site location: inferred from neighbouring sites
*R*[*F*^2^ > 2σ(*F*^2^)] = 0.043	H-atom parameters constrained
*wR*(*F*^2^) = 0.130	*w* = 1/[σ^2^(*F*_o_^2^) + (0.0676*P*)^2^ + 0.233*P*] where *P* = (*F*_o_^2^ + 2*F*_c_^2^)/3
*S* = 1.04	(Δ/σ)_max_ < 0.001
3626 reflections	Δρ_max_ = 0.26 e Å^−^^3^
250 parameters	Δρ_min_ = −0.22 e Å^−^^3^
0 restraints	

### Special details

**Table:**

Geometry. All esds (except the esd in the dihedral angle between two l.s. planes) are estimated using the full covariance matrix. The cell esds are taken into account individually in the estimation of esds in distances, angles and torsion angles; correlations between esds in cell parameters are only used when they are defined by crystal symmetry. An approximate (isotropic) treatment of cell esds is used for estimating esds involving l.s. planes.

### Fractional atomic coordinates and isotropic or equivalent isotropic displacement parameters (Å^2^)

**Table:**

	*x*	*y*	*z*	*U*_iso_*/*U*_eq_	
C1	0.10573 (17)	0.71389 (17)	1.15734 (9)	0.0393 (3)	
C2	0.21740 (18)	0.71690 (18)	1.24039 (10)	0.0430 (3)	
H2	0.3327	0.7711	1.2512	0.052*	
C3	0.15737 (19)	0.63927 (18)	1.30710 (10)	0.0435 (3)	
C4	−0.01447 (19)	0.55537 (18)	1.29072 (10)	0.0426 (3)	
C5	−0.12826 (18)	0.55865 (19)	1.20923 (10)	0.0446 (3)	
C6	−0.06846 (18)	0.63736 (18)	1.14315 (10)	0.0433 (3)	
H6	−0.1446	0.6392	1.0892	0.052*	
C7	0.4228 (3)	0.7414 (3)	1.41741 (15)	0.0841 (7)	
H7A	0.4901	0.7025	1.3701	0.126*	
H7B	0.4774	0.7334	1.4786	0.126*	
H7C	0.4144	0.8605	1.4220	0.126*	
C8	−0.1648 (3)	0.5368 (3)	1.41581 (15)	0.0809 (6)	
H8A	−0.2713	0.5483	1.3805	0.121*	
H8B	−0.1021	0.6501	1.4544	0.121*	
H8C	−0.1886	0.4634	1.4567	0.121*	
C9	−0.4173 (2)	0.4882 (3)	1.12169 (14)	0.0699 (5)	
H9A	−0.3869	0.4312	1.0624	0.105*	
H9B	−0.4172	0.6085	1.1248	0.105*	
H9C	−0.5302	0.4314	1.1248	0.105*	
C10	0.17536 (17)	0.78939 (18)	1.08679 (10)	0.0422 (3)	
H10	0.2915	0.8426	1.1020	0.051*	
C11	0.08636 (18)	0.78836 (19)	1.00250 (10)	0.0443 (3)	
H11	−0.0303	0.7369	0.9863	0.053*	
C12	0.16329 (17)	0.86397 (18)	0.93467 (10)	0.0426 (3)	
C13	0.07305 (17)	0.87319 (17)	0.84766 (10)	0.0392 (3)	
C14	0.16886 (19)	0.94308 (18)	0.78507 (11)	0.0441 (3)	
C15	−0.0952 (2)	0.88897 (19)	0.66628 (11)	0.0480 (3)	
C16	−0.11265 (18)	0.81213 (18)	0.81775 (10)	0.0417 (3)	
C17	0.1805 (3)	1.0066 (3)	0.63158 (14)	0.0735 (5)	
H17A	0.2704	1.1046	0.6673	0.110*	
H17B	0.1053	1.0413	0.5856	0.110*	
H17C	0.2307	0.9140	0.5986	0.110*	
C18	−0.3720 (2)	0.7703 (3)	0.69641 (14)	0.0674 (5)	
H18A	−0.3975	0.6558	0.6518	0.101*	
H18B	−0.4164	0.8507	0.6656	0.101*	
H18C	−0.4248	0.7668	0.7509	0.101*	
N1	0.08167 (17)	0.94549 (16)	0.69760 (9)	0.0479 (3)	
N2	−0.18496 (15)	0.82765 (16)	0.72844 (9)	0.0463 (3)	
O1	0.25706 (15)	0.63579 (17)	1.39048 (8)	0.0625 (3)	
O2	−0.06606 (16)	0.46212 (14)	1.35161 (8)	0.0573 (3)	
O3	−0.29489 (14)	0.47840 (17)	1.20048 (9)	0.0638 (3)	
O4	0.33028 (13)	0.92287 (18)	0.96038 (8)	0.0611 (3)	
H4	0.3661	0.9594	0.9180	0.092*	
O5	0.32765 (14)	1.00179 (16)	0.80691 (9)	0.0603 (3)	
O6	−0.16669 (18)	0.89585 (18)	0.58887 (8)	0.0693 (4)	
O7	−0.20868 (13)	0.75253 (17)	0.86397 (8)	0.0608 (3)	

### Atomic displacement parameters (Å^2^)

**Table:**

	*U*^11^	*U*^22^	*U*^33^	*U*^12^	*U*^13^	*U*^23^
C1	0.0395 (7)	0.0408 (7)	0.0361 (6)	0.0050 (5)	0.0078 (5)	0.0095 (5)
C2	0.0369 (7)	0.0482 (7)	0.0415 (7)	0.0046 (5)	0.0054 (5)	0.0117 (6)
C3	0.0455 (8)	0.0483 (7)	0.0378 (7)	0.0118 (6)	0.0055 (6)	0.0141 (6)
C4	0.0482 (8)	0.0422 (7)	0.0418 (7)	0.0091 (6)	0.0138 (6)	0.0164 (6)
C5	0.0393 (7)	0.0475 (7)	0.0455 (7)	0.0016 (6)	0.0092 (6)	0.0143 (6)
C6	0.0398 (7)	0.0524 (8)	0.0367 (7)	0.0038 (6)	0.0047 (5)	0.0151 (6)
C7	0.0596 (11)	0.1186 (17)	0.0669 (12)	−0.0029 (11)	−0.0179 (9)	0.0429 (12)
C8	0.0891 (14)	0.1140 (17)	0.0749 (13)	0.0458 (13)	0.0456 (11)	0.0575 (12)
C9	0.0363 (8)	0.0968 (14)	0.0761 (12)	−0.0053 (8)	0.0029 (8)	0.0393 (10)
C10	0.0353 (7)	0.0473 (7)	0.0413 (7)	0.0018 (5)	0.0082 (5)	0.0111 (6)
C11	0.0350 (7)	0.0537 (8)	0.0424 (7)	−0.0011 (6)	0.0078 (5)	0.0159 (6)
C12	0.0327 (6)	0.0493 (7)	0.0448 (7)	0.0012 (5)	0.0100 (5)	0.0142 (6)
C13	0.0354 (7)	0.0428 (7)	0.0409 (7)	0.0038 (5)	0.0119 (5)	0.0142 (5)
C14	0.0444 (8)	0.0436 (7)	0.0504 (8)	0.0080 (6)	0.0194 (6)	0.0186 (6)
C15	0.0592 (9)	0.0477 (8)	0.0417 (7)	0.0173 (6)	0.0125 (6)	0.0150 (6)
C16	0.0370 (7)	0.0455 (7)	0.0425 (7)	0.0053 (5)	0.0085 (5)	0.0138 (6)
C17	0.0860 (13)	0.0876 (13)	0.0673 (11)	0.0165 (11)	0.0381 (10)	0.0456 (10)
C18	0.0459 (9)	0.0906 (13)	0.0620 (10)	0.0097 (8)	−0.0030 (8)	0.0244 (9)
N1	0.0586 (8)	0.0482 (7)	0.0462 (7)	0.0127 (5)	0.0216 (6)	0.0224 (5)
N2	0.0426 (7)	0.0535 (7)	0.0431 (6)	0.0096 (5)	0.0064 (5)	0.0152 (5)
O1	0.0543 (7)	0.0855 (8)	0.0517 (6)	0.0084 (6)	0.0002 (5)	0.0352 (6)
O2	0.0710 (7)	0.0561 (6)	0.0581 (7)	0.0159 (5)	0.0247 (6)	0.0309 (5)
O3	0.0423 (6)	0.0871 (8)	0.0622 (7)	−0.0093 (5)	0.0059 (5)	0.0370 (6)
O4	0.0330 (5)	0.0924 (9)	0.0565 (7)	−0.0072 (5)	0.0047 (5)	0.0324 (6)
O5	0.0428 (6)	0.0774 (8)	0.0696 (7)	0.0017 (5)	0.0222 (5)	0.0362 (6)
O6	0.0839 (9)	0.0855 (9)	0.0461 (6)	0.0249 (7)	0.0099 (6)	0.0284 (6)
O7	0.0348 (5)	0.0905 (8)	0.0591 (7)	−0.0065 (5)	0.0078 (5)	0.0369 (6)

### Geometric parameters (Å, º)

**Table:**

C1—C2	1.3926 (19)	C10—C11	1.330 (2)
C1—C6	1.3964 (19)	C10—H10	0.9300
C1—C10	1.4592 (19)	C11—C12	1.4509 (19)
C2—C3	1.389 (2)	C11—H11	0.9300
C2—H2	0.9300	C12—O4	1.3108 (17)
C3—O1	1.3668 (17)	C12—C13	1.395 (2)
C3—C4	1.392 (2)	C13—C14	1.4414 (18)
C4—O2	1.3713 (17)	C13—C16	1.4545 (19)
C4—C5	1.400 (2)	C14—O5	1.2484 (18)
C5—O3	1.3613 (17)	C14—N1	1.3728 (19)
C5—C6	1.3854 (19)	C15—O6	1.2115 (19)
C6—H6	0.9300	C15—N2	1.377 (2)
C7—O1	1.403 (2)	C15—N1	1.388 (2)
C7—H7A	0.9600	C16—O7	1.2171 (17)
C7—H7B	0.9600	C16—N2	1.3949 (18)
C7—H7C	0.9600	C17—N1	1.4651 (19)
C8—O2	1.397 (2)	C17—H17A	0.9600
C8—H8A	0.9600	C17—H17B	0.9600
C8—H8B	0.9600	C17—H17C	0.9600
C8—H8C	0.9600	C18—N2	1.464 (2)
C9—O3	1.427 (2)	C18—H18A	0.9600
C9—H9A	0.9600	C18—H18B	0.9600
C9—H9B	0.9600	C18—H18C	0.9600
C9—H9C	0.9600	O4—H4	0.8200
			
C2—C1—C6	119.47 (12)	C10—C11—H11	118.6
C2—C1—C10	118.69 (12)	C12—C11—H11	118.6
C6—C1—C10	121.83 (12)	O4—C12—C13	120.83 (12)
C3—C2—C1	120.32 (13)	O4—C12—C11	114.45 (12)
C3—C2—H2	119.8	C13—C12—C11	124.71 (12)
C1—C2—H2	119.8	C12—C13—C14	118.37 (12)
O1—C3—C2	124.48 (13)	C12—C13—C16	122.27 (12)
O1—C3—C4	115.30 (12)	C14—C13—C16	119.35 (13)
C2—C3—C4	120.22 (13)	O5—C14—N1	118.50 (12)
O2—C4—C3	119.14 (13)	O5—C14—C13	122.93 (14)
O2—C4—C5	121.34 (13)	N1—C14—C13	118.57 (13)
C3—C4—C5	119.39 (12)	O6—C15—N2	122.00 (15)
O3—C5—C6	124.28 (13)	O6—C15—N1	121.53 (15)
O3—C5—C4	115.49 (12)	N2—C15—N1	116.47 (13)
C6—C5—C4	120.22 (13)	O7—C16—N2	118.09 (13)
C5—C6—C1	120.23 (13)	O7—C16—C13	126.00 (13)
C5—C6—H6	119.9	N2—C16—C13	115.90 (12)
C1—C6—H6	119.9	N1—C17—H17A	109.5
O1—C7—H7A	109.5	N1—C17—H17B	109.5
O1—C7—H7B	109.5	H17A—C17—H17B	109.5
H7A—C7—H7B	109.5	N1—C17—H17C	109.5
O1—C7—H7C	109.5	H17A—C17—H17C	109.5
H7A—C7—H7C	109.5	H17B—C17—H17C	109.5
H7B—C7—H7C	109.5	N2—C18—H18A	109.5
O2—C8—H8A	109.5	N2—C18—H18B	109.5
O2—C8—H8B	109.5	H18A—C18—H18B	109.5
H8A—C8—H8B	109.5	N2—C18—H18C	109.5
O2—C8—H8C	109.5	H18A—C18—H18C	109.5
H8A—C8—H8C	109.5	H18B—C18—H18C	109.5
H8B—C8—H8C	109.5	C14—N1—C15	123.97 (12)
O3—C9—H9A	109.5	C14—N1—C17	118.57 (14)
O3—C9—H9B	109.5	C15—N1—C17	117.44 (14)
H9A—C9—H9B	109.5	C15—N2—C16	125.68 (13)
O3—C9—H9C	109.5	C15—N2—C18	116.73 (13)
H9A—C9—H9C	109.5	C16—N2—C18	117.58 (13)
H9B—C9—H9C	109.5	C3—O1—C7	117.14 (13)
C11—C10—C1	125.35 (13)	C4—O2—C8	116.71 (13)
C11—C10—H10	117.3	C5—O3—C9	117.32 (12)
C1—C10—H10	117.3	C12—O4—H4	109.5
C10—C11—C12	122.86 (13)		
			
C6—C1—C2—C3	2.1 (2)	C16—C13—C14—N1	−1.7 (2)
C10—C1—C2—C3	−177.07 (13)	C12—C13—C16—O7	2.1 (2)
C1—C2—C3—O1	−179.95 (13)	C14—C13—C16—O7	−179.05 (15)
C1—C2—C3—C4	1.0 (2)	C12—C13—C16—N2	−179.24 (12)
O1—C3—C4—O2	−6.8 (2)	C14—C13—C16—N2	−0.35 (19)
C2—C3—C4—O2	172.34 (13)	O5—C14—N1—C15	−178.00 (13)
O1—C3—C4—C5	177.24 (13)	C13—C14—N1—C15	1.9 (2)
C2—C3—C4—C5	−3.6 (2)	O5—C14—N1—C17	3.8 (2)
O2—C4—C5—O3	6.2 (2)	C13—C14—N1—C17	−176.34 (14)
C3—C4—C5—O3	−177.92 (13)	O6—C15—N1—C14	179.01 (14)
O2—C4—C5—C6	−172.77 (13)	N2—C15—N1—C14	0.1 (2)
C3—C4—C5—C6	3.1 (2)	O6—C15—N1—C17	−2.7 (2)
O3—C5—C6—C1	−178.84 (14)	N2—C15—N1—C17	178.30 (14)
C4—C5—C6—C1	0.0 (2)	O6—C15—N2—C16	178.65 (14)
C2—C1—C6—C5	−2.7 (2)	N1—C15—N2—C16	−2.4 (2)
C10—C1—C6—C5	176.53 (13)	O6—C15—N2—C18	0.1 (2)
C2—C1—C10—C11	176.58 (14)	N1—C15—N2—C18	179.10 (14)
C6—C1—C10—C11	−2.6 (2)	O7—C16—N2—C15	−178.70 (14)
C1—C10—C11—C12	−179.27 (13)	C13—C16—N2—C15	2.5 (2)
C10—C11—C12—O4	4.4 (2)	O7—C16—N2—C18	−0.2 (2)
C10—C11—C12—C13	−176.76 (14)	C13—C16—N2—C18	−179.01 (14)
O4—C12—C13—C14	2.3 (2)	C2—C3—O1—C7	10.7 (2)
C11—C12—C13—C14	−176.48 (13)	C4—C3—O1—C7	−170.18 (17)
O4—C12—C13—C16	−178.81 (13)	C3—C4—O2—C8	106.41 (19)
C11—C12—C13—C16	2.4 (2)	C5—C4—O2—C8	−77.7 (2)
C12—C13—C14—O5	−2.9 (2)	C6—C5—O3—C9	−4.9 (2)
C16—C13—C14—O5	178.20 (13)	C4—C5—O3—C9	176.16 (15)
C12—C13—C14—N1	177.25 (12)		

### Hydrogen-bond geometry (Å, º)

**Table:**

*D*—H···*A*	*D*—H	H···*A*	*D*···*A*	*D*—H···*A*
O4—H4···O5	0.82	1.74	2.4841 (15)	150
C11—H11···O7	0.93	2.16	2.8044 (18)	125
C8—H8*B*···O6^i^	0.96	2.60	3.341 (3)	135
C9—H9*C*···O7^ii^	0.96	2.42	3.3694 (19)	170

Symmetry codes: (i) *x*, *y*, *z*+1; (ii) −*x*−1, −*y*+1, −*z*+2.

## References

[bb1] Bojarski, J. T., Mokrosz, J. L., Bartoń, H. J. & Paluchowska, M. H. (1985). *Advances in Heterocyclic Chemistry*, Vol. 38, edited by A. Katritzky. New York: Academic Press.

[bb2] Bruker (2012). *APEX2* and *SAINT*. Bruker AXS Inc., Madison, Wisconsin, USA.

[bb3] Bruker (2015). *SADABS*. Bruker AXS Inc., Madison, Wisconsin, USA.

[bb4] Bruno, I. J., Cole, J. C., Kessler, M., Luo, J., Motherwell, W. D. S., Purkis, L. H., Smith, B. R., Taylor, R., Cooper, R. I., Harris, S. E. & Orpen, A. G. (2004). *J. Chem. Inf. Comput. Sci.* **44**, 2133–2144. 10.1021/ci049780b 15554684

[bb5] Dolomanov, O. V., Bourhis, L. J., Gildea, R. J., Howard, J. A. K. & Puschmann, H. (2009). *J. Appl. Cryst.* **42**, 339–341.

[bb6] Farrugia, L. J. (2012). *J. Appl. Cryst.* **45**, 849–854.

[bb7] Gorovoy, A. S., Guyader, D. & Lejon, T. (2014). *Synth. Commun.* **44**, 1296–1300.

[bb8] Goswami, P. & Das, B. (2009). *Tetrahedron Lett.* **50**, 897–900.

[bb9] Groom, C. R., Bruno, I. J., Lightfoot, M. P. & Ward, S. C. (2016). *Acta Cryst.* B**72**, 171–179. 10.1107/S2052520616003954 PMC4822653 27048719

[bb10] Hirshfeld, F. L. (1977). *Theor. Chim. Acta*, **44**, 129–138.

[bb11] Klikar, M., Bureš, F., Pytela, O., Mikysek, T., Padělková, Z., Barsella, A., Dorkenoo, K. & Achelle, S. (2013). *New J. Chem.* **37**, 4230–4240.

[bb12] Macrae, C. F., Bruno, I. J., Chisholm, J. A., Edgington, P. R., McCabe, P., Pidcock, E., Rodriguez-Monge, L., Taylor, R., van de Streek, J. & Wood, P. A. (2008). *J. Appl. Cryst.* **41**, 466–470.

[bb13] Mahmudov, K. T., Kopylovich, M. N., Maharramov, A. M., Kurbanova, M. M., Gurbanov, A. V. & Pombeiro, A. J. L. (2014). *Coord. Chem. Rev.* **265**, 1–37.

[bb14] McKinnon, J. J., Jayatilaka, D. & Spackman, M. A. (2007). *Chem. Commun.* pp. 3814–3816. 10.1039/b704980c 18217656

[bb15] McKinnon, J. J., Spackman, M. A. & Mitchell, A. S. (2004). *Acta Cryst.* B**60**, 627–668. 10.1107/S0108768104020300 15534375

[bb16] Patrick, G. L. (2009). In *An Introduction to Medicinal Chemistry*, p. 752. Oxford University Press.

[bb17] Seifert, S., Seifert, A., Brunklaus, G., Hofmann, K., Rüffer, T., Lang, H. & Spange, S. (2012). *New J. Chem.* **36**, 674–684.

[bb18] Sheldrick, G. M. (2008). *Acta Cryst.* A**64**, 112–122. 10.1107/S0108767307043930 18156677

[bb19] Sheldrick, G. M. (2015). *Acta Cryst.* C**71**, 3–8.

[bb20] Spackman, M. A. & McKinnon, J. J. (2002). *CrystEngComm*, **4**, 378–392.

